# Neoglycosylated Collagen: Effect on Neuroblastoma F-11 Cell Lines

**DOI:** 10.3390/molecules25194361

**Published:** 2020-09-23

**Authors:** Antonella Sgambato, Valentina Pastori, Laura Russo, Simone Vesentini, Marzia Lecchi, Laura Cipolla

**Affiliations:** 1 Department of Biotechnology and Biosciences, University of Milano—Bicocca, Piazza della Scienza 2, 20126 Milano, Italy; antonella.sgambato@unimib.it (A.S.); valentina.pastori@unimib.it (V.P.); laura.russo@unimib.it (L.R.); 2 Department of Electronics, Information and Bioengineering, Politecnico di Milano, via Camillo Golgi 39, 20133 Milano, Italy; simone.vesentini@polimi.it

**Keywords:** collagen, biomaterials, carbohydrates, chondroitin sulfate, F-11 cell line, sialic acid, glucose, galactose

## Abstract

The regeneration of the nervous system is a challenging task. Currently, regenerative medicine approaches that exploit nature-inspired cues are being studied and hold great promise. The possibility to use protein-based matrices functionalized with small oligo- and monosaccharides is of interest since these can be finely tuned to better mimic the native environment. Collagen has been selected as a promising material that has the potential to be further tailored to incorporate carbohydrates in order to drive cell behavior towards neuroregeneration. Indeed, the grafting of carbohydrates to collagen 2D matrices is proved to enhance its biological significance. In the present study, collagen 2D matrices were grafted with different carbohydrate epitopes, and their potential to drive F-11 neuroblastoma cells towards neuronal differentiation was evaluated. Collagen functionalized with α-glucosides was able to differentiate neuroblastoma cells into functional neurons, while sialyl α-(2→6)-galactosides stimulated cell proliferation.

## 1. Introduction

Nervous system repair and/or function restoration after injury, traumas or neurological disorders, such as neurodegenerative diseases, is still a challenging and unsolved problem. The main approaches currently being proposed include pharmacological treatments (limited by drug delivery issues [1]) that result in the slowing of disease progression [2] and regenerative medicine approaches [3,4], all of which are flanked by the improvement of our knowledge of the nervous system.

Regenerative medicine approaches often rely on the combination of biomaterials able to support tissue regeneration, cells (autologous, xeno- or autografts) and signaling cues able to drive cell responses towards tissue regeneration. Collagen, the most abundant protein in the human body [5], is emerging as a suitable biomaterial for regenerative medicine [6], including for nervous system repair [7,8,9], due to its biocompatibility, its biodegradability and its mechanical properties matching the nervous tissue, in the form of hydrogels [10]. In addition, interest in the role of collagen in the nervous system has been increasing over recent decades, given the role of collagen in signal transduction and nerve regeneration [11,12].

**Figure 1:**
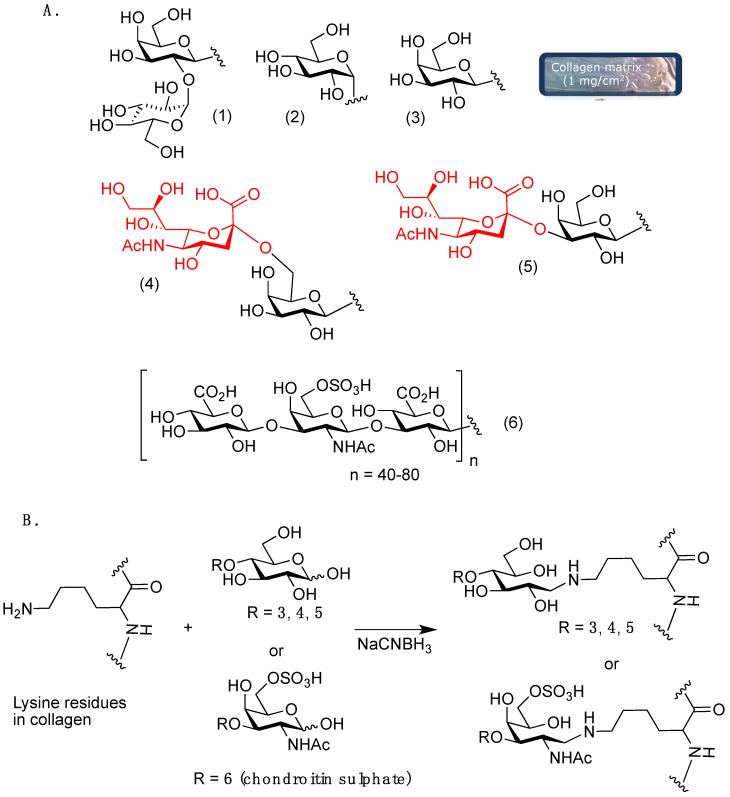
(**A**) Carbohydrate epitopes grafted to collagen matrices: (1) the native collagen glycosylation motif, (2) α-glucosides, (3) β-galactosides, (4) sialyl α-(2→6)-galactoside, (5) sialyl α-(2→3)-galactoside, (6) chondroitin sulphate. (**B**) Grafting chemistry for collagen matrix functionalization.

Besides collagen, carbohydrates play a significant role as key signaling biochemical cues and can be considered useful molecules in regenerative medicine approaches [13], including nervous tissue regeneration [14]. The ability of carbohydrates to drive neuronal cell behavior has been shown in previous works [15,16]. Moreover, collagen itself is constitutively glycosylated at hydroxylysine residues with the disaccharide α-(1→2)-glucosylgalactoside **1** (Figure 1) [17]. Thus, new synthetic glycosylated collagen matrices grafted with relevant saccharides involved in nervous system regulation and development have been prepared and evaluated.

## 2. Results and Discussion

We previously showed that carbohydrate epitopes grafted to collagen are able to stimulate specific cell responses, depending on the combination of epitope and cell line [16,18,19,20].

Since carbohydrates are deeply involved in neuronal development, we explored F-11 cell behavior in the presence of collagen matrices grafted with different carbohydrate epitopes (Figure 1A).

Within this framework, we showed that α-glucosides grafted to collagen 2D matrices (**2**, Figure 1A) are able to drive neuroblastoma F-11 cell lines to differentiate into functional neurons [16].

In this work, galactosides (**3**), sialyl α-(2→6)-galactoside (**4**), sialyl α-(2→3)-galactoside (**5**) and chondroitin sulphate (**6**) were selected for collagen 2D matrices grafting.

Galactosides are found in galactosylceramides present in myelin, and in the myelin sheath. Studies suggest that galactosides are relevant for the myelination process and/or in myelin function [21]. Galactosides were included in polymeric materials grafted with small carbohydrate epitopes, together with L-fucose, *N*-acetylglucosamine Fuc-α-(1→2)-Gal and Fuc-α-(1→3)-GlcNAc; their potential to stimulate a neuronal response toward hippocampal neurons [22] was evaluated, and few of them were able to stimulate neurite outgrowth.

Sialyl α-(2→3)-galactoside, sialyl α-(2→6)-galactoside and chondroitin sulphate grafted to collagen were also investigated. Sialic acids are involved in several biological functions, including neuronal development, especially in the form of gangliosides, a lipid class; they are usually found as terminal moieties linked to D-galactose residues through α-(2→6)- or α-(2→3)-linkages (**4** and **5**, Figure 1A). α-(2→3)-linked sialosides are the most common. The interaction of the sialic acid-binding immunoglobulin-like lectins (Siglec) [23] with sialosides exposed by gangliosides on the axon regulates the formation and maintenance of the myelin-axon organization and is involved in demyelinating disorders, such as multiple sclerosis; thus, sialosides may provide new opportunities in nervous system tissue engineering after central nervous system (CNS) injury or disease, promoting axon regeneration. To the best of our knowledge, no sialic acid grafted biomaterials have been proposed for neural tissue regeneration; however, we showed that collagen grafted with α-(2→3)-galactosides upregulate genes related to osteogenesis, while sialyl α-(2→6)-galactosides influence chondrogenesis marker expression [18].

Chondroitin sulphate (CS) is an abundant component of the brain extracellular matrix (ECM), and together with another glycosaminoglycan, hyaluronan, it forms macromolecular aggregates in the perineuronal nets, which cover cell bodies and the proximal part of neurites of several neuronal populations. CS is usually linked to proteins, affording the so-called proteoglycans. Soluble chondroitin sulphate proteoglycan (CSPG) and substratum-bound CSPG have different effects on neuronal growth cone behavior. Substratum-bound CSPG inhibits neurite outgrowth and the rate of neurite elongation of dorsal root ganglion (DRG) neurons [24]. CS is largely used in biomaterial design for bone, cartilage and neural tissue regeneration [25]. In addition, biomaterials grafted with chondroitin sulphate-derived disaccharides have been proposed, and researchers have shown that biomaterials grafted with chondroitin sulphate motifs constitute interesting approaches towards neural cell differentiation and neural tissue regeneration [26]. Moreover, we recently synthesised a chimeric proteoglycan composed of collagen and CS [27], mimicking the covalent link between the protein and the polysaccharide in proteoglycans and guaranteeing the native orientation of the glycan chain protruding from the protein backbone. Here we propose the evaluation of this chimeric proteoglycan as a biomaterial for neural tissue regeneration.

Thus, carbohydrate motifs **2**–**6** (Figure 1A) were grafted to collagen matrices, taking advantage of the complementary reactivity of lysine side-chain amino groups and the reducing end of suitable saccharides (Figure 1B), exposing the desired carbohydrate epitopes **2**–**6**: the reaction in reducing conditions by NaCNBH_3_ regioselectivity affords a stable amine linkage between collagen and the saccharidic moieties.

The biological activity of neoglycosylated collagen matrices was evaluated with F-11 cells, a neuroblastoma cell line which proliferates in 10% serum medium but expresses electrophysiological properties of dorsal root ganglion (DRG) neurons under appropriate culture conditions [28]. F-11 was used as the cell line of choice because we previously demonstrated that it is able to generate functional neurons by its maintenance on neoglucosylated collagen matrices [16].

**Figure 2:**
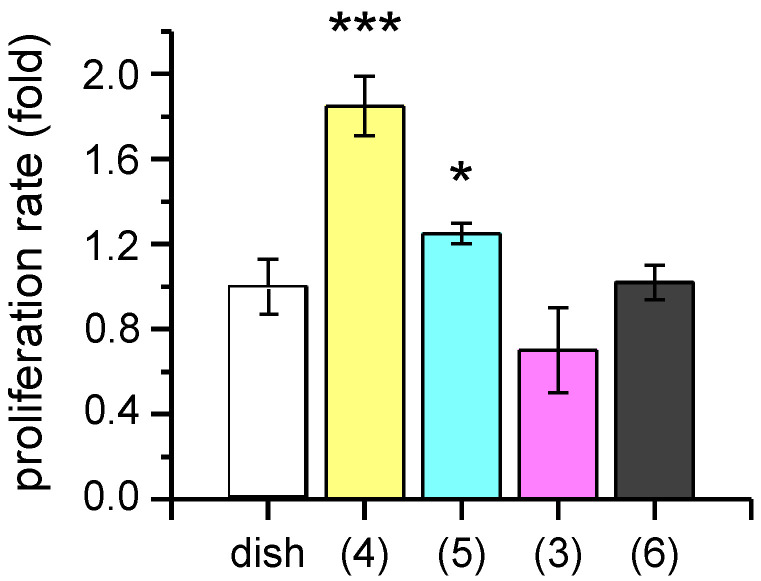
Proliferation rate of F-11 cells seeded and maintained for seven days on different neoglycosylated collagen matrices: (**4**) sialyl α-(2→6)-galactoside, (**5**) sialyl α-(2→3)-galactoside, (**3**) β-galactoside, (**6**) chondroitin sulphate. * *p* < 0.05, *** *p* < 0.001.

First, cell proliferation was assessed in order to evaluate biocompatibility of neoglycosylated collagen matrices. F-11 cells maintained for seven days on sialyl α-(2→6)-galactoside (**4**) and sialyl α-(2→3)-galactoside (**5**) grafted to collagen matrices showed, respectively, an 85 ± 14% (*p* < 0.001) and 25 ± 5% (*p* < 0.05) increase in proliferation rate, compared to cells grown on Petri dish. On the contrary, β-galactoside (**3**) and chondroitin sulphate (**6**) collagen matrices did not behave differently from control (Figure 2). Taken together, these data suggest that sialic acids specifically sustain F-11 cell proliferation, while galactosides and chondroitin sulphate do not. It has been shown that gangliosides expressed in neural stem cells and neuroblastomas are different from those present in mature neurons; they are able to interact with the epidermal growth factor (EGF) receptor, allowing proliferation and the maintenance of the undifferentiated state (in neural stem cells) and contributing to the oncogenic properties (in neuroblastomas). In particular, simpler gangliosides featured by a single sialic acid residue are more expressed on neuroblastoma cells and are possibly related to cell proliferation [29]. Since F-11 is a neuroblastoma cell line, this could provide a possible explanation for the proliferative effect of sialic acids we observed. Interestingly, cell proliferation was sensitive to sialoside regiochemistry ((2→6) versus (2→3)).

**Figure 3:**
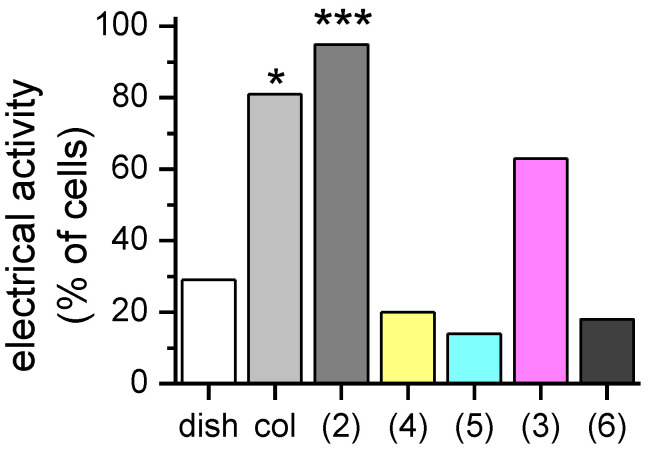
Percentage of F-11 cells endowed with electrical activity on different neoglycosylated collagen matrices: col, pristine collagen; (**2**) α-glucosides, (**4**) sialyl α-(2→6)-galactoside, (**5**) sialyl α-(2→3)-galactoside, (**3**) β-galactoside, (**6**) chondroitin sulphate. Statistical analysis was performed using the Chi-squared test. Significance was set for *p* < 0.05. * *p* < 0.05, *** *p* < 0.001.

Since our previous work showed a higher percentage of F-11 cells with neuron-like morphological and functional properties when plated on α-glucoside collagen matrices than when plated on pristine collagen or a Petri dish [16], in the new samples we also investigated cell electrical activity. Patch-clamp recordings demonstrated that, conversely to α-glucosides, these sugar epitopes did not provide a differentiating pressure on F-11 cells. In fact, the percentage of cells able to generate action potentials (the hallmark of neurons) did not change compared to the Petri dish group, but it was reduced compared to the pristine and α-glucoside collagen matrices (**2**). In particular, the percentages of cells showing electrical activity on sialyl α-(2→3)-galactoside (**5**), sialyl α-(2→6)-galactoside (**4**) or chondroitin sulphate (**6**) collagen matrices were, respectively, 20%, 14% and 18%. Only the β-galactoside collagen matrix (**3**) slightly increased this percentage compared to the Petri dish group (63% of cells on β-galactoside collagen versus 29% on Petri dish); however, the difference was not significant (*p* > 0.05) (Figure 3). The inability of β-galactosides (**3**) to drive cell behavior is in agreement with previous results [22] showing that biomaterials functionalized with galactosides failed to promote neuronal outgrowth.

## 3. Materials and Methods 

### 3.1. General Methods

Solvents and reagents were purchased from Sigma-Aldrich (Saint Louis, MO, USA) and used without further purification. Milli-Q water (mQ) was obtained with 18.2 MΩ cm at a 25 °C purity. Chondroitin sulphate sodium salt (from shark cartilage, CAS number 12678-07-8) and sialyllactosides were purchased from Carbosynth Ltd. (Newbury, UK).

### 3.2. Matrices Preparation

#### 3.2.1. Pristine Collagen Matrices

Collagen matrices were prepared from insoluble Collagen Type I from bovine Achilles tendon (Sigma-Aldrich, Saint Louis, MO, USA, catalogue no. C9879) by the solvent casting method [30]. Briefly, collagen powder was suspended in acetic acid 0.5 M at 40 °C under stirring. After 4 h, the suspension was homogenized with a mixer for 2 min at maximum speed. After removal of the aggregates, 40 mL of collagen solution was poured into an 8.5 × 12.5 cm^2^ culture multiwell lid, and the solvent evaporated in the fume hood over 48 h. The resulting collagen matrices appeared as transparent films (1 mg/cm^2^). The film thickness was in the 6−7 μm range, as determined by SEM [18].

#### 3.2.2. Carbohydrate-Grafted Collagen Matrices

Carbohydrate-grafted matrices were prepared and characterised as previously described. Grafting chemistry was based on a reductive amination reaction between lysine amino groups of collagen and the carbonyl reducing end of lactose [31], sialyllactosides [18] and chondroitin sulphate [27] in the presence of NaCNBH_3_ as reducing agent.

β-Galactose-grafted collagen [31]: collagen matrix (80 mg, 12 × 7 cm) was immersed in 20 mL of a 0.06 M lactose citrate buffer solution (pH 6.00), and a 0.03 M NaCNBH_3_/citrate buffer was added and reacted overnight. The collagen matrix was washed with 20 mL of Milli-Q (mQ) (3 × 20 min), and then finally washed with 20 mL of ethanol (20 min).

Sialyl galactosides-grafted collagen [18]: collagen matrices (80 mg, 12 × 7 cm) were immersed in 20 mL of either a 0.006 M 6′-sialyllactose or a 3′-sialyllactose citrate buffer solution (pH 6.00), and a 0.003 M NaCNBH_3_/citrate buffer was added and reacted overnight. The collagen matrix was washed with 20 mL of Milli-Q (mQ) (3 × 20 min), and then finally washed with 20 mL of ethanol (20 min).

Chondroitin sulphate-grafted collagen [27]: a collagen matrix (1 mg, 2 × 1 cm) was immersed in 2 mL of a 1.5 mM chondroitin sulphate citrate buffer solution (pH 6.00), and a 0.75 mM NaCNBH_3_/citrate buffer was added and reacted overnight. The collagen matrix was recovered and washed with 2 mL of HCl 0.1 M for 10 min, followed by 2 mL of NaOH 0.1 M for 10 min, 2 mL of Milli-Q (mQ) water (3 × 20 min) and finally 2 mL of ethanol for 10 min.

### 3.3. Cell Culture

F-11 cells, a hybrid of N18TG2 mouse neuroblastoma cell line and dorsal root ganglion (DRG) neurons, were seeded at 60,000 cells/35 mm dish and maintained on the different neoglycosylated collagen matrices for 7 days without splitting. The cells were cultured in Dulbecco’s modified Eagle’s medium (DMEM, Sigma-Aldrich, Saint Louis, MO, USA), supplemented with 2 mM glutamine (Sigma-Aldrich, Saint Louis, MO, USA) and 10% fetal bovine serum (FBS, Sigma-Aldrich, Saint Louis, MO, USA) and incubated at 37 °C in a humidified atmosphere with 5% CO_2_. Cells grown on Petri dish and/or on pristine collagen were used as control.

The culture medium was refreshed twice per week. Seven days after seeding, proliferation assay and functional analysis were performed.

#### 3.3.1. Proliferation Assay and Functional Analysis by Patch-Clamp Recordings

The proliferation rate was evaluated 7 days after seeding, counting cells by microscopy observations using a Bürker chamber. For each matrix, 3–9 samples were investigated. Electrophysiological analysis was performed by the patch-clamp technique in the whole-cell configuration. Recordings were acquired by the pClamp 8.2 software (Molecular Devices, LLC., San Jose, CA, USA) and the MultiClamp 700A amplifier (Axon Instruments, Molecular Devices, LLC., San Jose, CA, USA) in the current-clamp mode.

Before starting the experiment, the culture medium was replaced with a standard extracellular solution, which was also bath applied during recordings, and contained the following (mM): NaCl 135, KCl 2, CaCl_2_ 2, MgCl_2_ 2, hepes 10, glucose 5 and pH 7.4. The standard pipette solution contained the following (mM): potassium aspartate 130, NaCl 10, MgCl_2_ 2, CaCl_2_ 1.3, EGTA 10, hepes 10 and pH 7.3.

The parameters we extracted from the recordings were the resting membrane potential (V_rest_) and the percentage of cells able to generate spontaneous or induced action potentials. The electrical activity was evoked by hyperpolarizing the V_rest_ at approximately −75 mV and subsequently depolarizing the membrane with 600 ms long current pulses. The depolarization peaks were considered action potentials when they were higher than 0 mV. Patch-clamp experiments were performed on 4–9 samples per each matrix, and the recorded cells were *n* = 21 for the dish, *n* = 12 for native collagen, *n* = 24 for (**2**), *n* = 10 for (**4**), *n* = 7 for (**5**), *n* = 8 for (**3**) and *n* = 13 for (**6**).

#### 3.3.2. Statistical Analysis

For the analysis, Origin 8 (Microcal Inc., Northampton, MA, USA) and Excel were used. Data are presented as mean ± SEM. Statistical evaluations were obtained using one-way analysis of variance (ANOVA), followed by the Tukey post hoc test and the Chi-squared test. Values were considered statistically significant if *p* < 0.05.

## 4. Conclusions

Collagen, an interesting biomaterial for regenerative medicine approaches in neuroregeneration, was grafted with different carbohydrates known to be involved in neuronal development and functions. The F-11 neuroblastoma cell line was used as model cells to assess carbohydrate potential in neuroregeneration: α-glucosides grafted to collagen were the only carbohydrate epitopes able to drive neuroblastoma cells to differentiate into functional neurons. On the contrary, sialyl galactosides increased cell proliferation, while β-galactoside and chondroitin sulphate did not show any significant influence on cell behavior.
